# Centrality Measures in Residue Interaction Networks to Highlight Amino Acids in Protein–Protein Binding

**DOI:** 10.3389/fbinf.2021.684970

**Published:** 2021-06-18

**Authors:** Guillaume Brysbaert, Marc F. Lensink

**Affiliations:** Univ. Lille, CNRS UMR 8576 - UGSF - Unité de Glycobiologie Structurale et Fonctionnelle, Lille, France

**Keywords:** residue interaction networks, centrality, protein structure networks, structural bioinformatics, protein binding, protein–protein interaction, protein complexes, protein assemblies

## Abstract

Residue interaction networks (RINs) describe a protein structure as a network of interacting residues. Central nodes in these networks, identified by centrality analyses, highlight those residues that play a role in the structure and function of the protein. However, little is known about the capability of such analyses to identify residues involved in the formation of macromolecular complexes. Here, we performed six different centrality measures on the RINs generated from the complexes of the SKEMPI 2 database of changes in protein–protein binding upon mutation in order to evaluate the capability of each of these measures to identify major binding residues. The analyses were performed with and without the crystallographic water molecules, in addition to the protein residues. We also investigated the use of a weight factor based on the inter-residue distances to improve the detection of these residues. We show that for the identification of major binding residues, closeness, degree, and PageRank result in good precision, whereas betweenness, eigenvector, and residue centrality analyses give a higher sensitivity. Including water in the analysis improves the sensitivity of all measures without losing precision. Applying weights only slightly raises the sensitivity of eigenvector centrality analysis. We finally show that a combination of multiple centrality analyses is the optimal approach to identify residues that play a role in protein–protein interaction.

## Introduction

Protein structures inherently contain an abundance of information, the extraction of which is often performed in order to correlate feature with function. Many complementary approaches have been developed to this end, based on a protein’s primary to quaternary structure. At the primary—sequence—level, the approaches typically rely on comparison to annotated sequences. This can lead to reliable predictions of backbone flexibility, secondary structure with phi and psi angles, or even three-dimensional structural modeling (Kryshtafovych et al., 2019). Indeed, the availability of the three-dimensional structure of a protein allows one to go a step further and extract information related to its function. The positioning of amino acids and orientation of side chains provide valuable information for the selection of residues for mutagenesis or to design drugs. In addition to this, a protein rarely works alone and often engages in macromolecular complex formation in order for it to execute its biological function. These complexes include homodimers at their simplest level but often reach dozens of molecules. The wwPDB [(Berman et al., 2003)^1^] contains the experimentally resolved structures of proteins, occasionally in complexes with their binding partners. In the absence of such information, molecular docking tools have been developed to predict the interaction between protein partners with increasing reliability (Lensink et al., 2019; Lensink et al., 2020).

Derived from structures of proteins, a graph approach was developed roughly 20 years ago (Kannan and Vishveshwara, 1999; Vendruscolo et al., 2002). This approach consists of generating a network (or graph) of residues from their contacts in a PDB structure. These graphs are named residue interaction networks (RINs) or residue interaction graphs (“Amino Acid” may be used instead of “Residue”) but are also called protein structure networks or protein structure graphs (Amitai et al., 2004; Doncheva et al., 2011; Greene, 2012; Felline et al., 2020), or even protein contact networks (Di Paola et al., 2015). They show nodes as residues and edges as interaction detected between them. These networks can be generated from a single structure or a complex as long as the chains are available in the same PDB file, for example, Brysbaert et al. (2018), de Ruyck et al. (2018), and Vishveshwara et al. (2009). Although it is a simplified representation of a protein structure, it brings with it the availability of a myriad of tools developed for network analysis. In the context of proteins, centrality calculations have been shown to be effective in the identification of biologically relevant residues and many centrality measures currently exist (Vacic et al., 2010; Doncheva et al., 2012; Brysbaert et al., 2017; Chakrabarty and Parekh, 2016; Newaz et al., 2020). But among the more widely used ones, we find betweenness, closeness, residue centrality analysis, and the degree (Amitai et al., 2004; del Sol et al., 2006; Liu and Hu, 2011; Hu et al., 2014; Jiao and Ranganathan, 2017). More recently, the eigenvector centrality started to gain popularity (Chakrabarty and Parekh, 2016). Choosing a centrality measure is not trivial largely due to the lack of scientific consensus as to which measure performs better than another, particularly in relation to the identification of the more relevant binding residues. In this protein–protein interaction (PPI) context, del Sol and O’Meara showed that the betweenness centrality is a good measure to identify hot spots on a set of 18 protein complexes (del Sol and O'Meara, 2005). They showed that high betweenness residues tend to be located in regions where experimentally validated hot spots are present, emphasizing the interest in using these types of measures in a PPI context. However, their study was limited to the betweenness centrality measure and to 18 complexes, although of various types. Some works also used the betweenness centrality and others used the closeness or degree (Di Paola et al., 2015; Stetz and Verkhivker, 2017; Brysbaert et al., 2018; de Ruyck et al., 2018) but always in specific PPI contexts and an evaluation of these statistical metrics on a larger set is missing.

Here, we aim to fill two voids: we first evaluate the capability of centrality measures to highlight residues that are essential for the binding of two proteins on a large set, and second, we compare these measures to show which ones are the best to use. For this purpose, we used the SKEMPI 2 database, which is a benchmark set of changes in protein–protein binding energy, in kinetics, and in thermodynamics upon mutation (Jankauskaitė et al., 2019). We generated one RIN for each complex in the dataset and ran 6 centrality measures: the 5 cited above and the well-known Google PageRank (Brin and Page, 1998). We then evaluated the efficiency of each measure to find the residues that disrupt the binding upon mutation. We also considered water molecules and a residue–residue distance weight to assess their relevance in the centrality analysis. We further evaluated the benefit of combining the results of centrality measures with unions and intersections in order to improve the results. And we finally evaluated which measure is preferred for use in the context of the location of the residues of interest.

## Materials and Methods

### Dataset

The SKEMPI version 2 dataset of April 8th, 2018 (Jankauskaitė et al., 2019) was used for the evaluation of the centrality measures, which represents a set of 7,085 mutations. The majority of the dataset consists of single-point mutations (5,112), while the remainder represents sets of mutations (1,973). We considered only single-point mutations so that we may associate a specific mutation to a change in binding. For each mutation, a binding free-energy difference |∆∆G_binding_| was computed from the affinity values listed in the SKEMPI 2 dataset. We considered both positive as well as negative values, hence including mutations that lead to both an enhanced or diminished binding. Since residue centrality analyses are residue-centric, we kept only the mutation associated with the largest |∆∆G_binding_| to avoid counting the residue multiple times if it has been mutated to multiple other residues. We thus ignored the lower values of |∆∆G_binding_|, the maximum being sufficient to evaluate if a residue is disrupting or not. This results in a total of 3,039 residues subjected to mutation that were used for analysis. These mutations are found in 323 complexes, which represents 93.6% of the total SKEMPI 2 dataset (345 PDBs in total), ensuring sufficient conservation of the heterogeneity of the full set (for a description of the full set, see Jankauskaitė et al. (2019)).

We classified the residues according to their |∆∆G_binding_|, considering 3 thresholds: 0.592, 1.184, and 2 kcal/mol; a residue was considered as major if its |∆∆G_binding_| was superior or equal to the threshold. The 2 kcal/mol threshold was chosen because ∆∆G_binding_ ≥ 2 kcal/mol is a well-accepted definition for hot spots (Moreira et al., 2007b; Moreira et al., 2017a), that is, sites which show such a variation in binding free energy upon mutation to alanine. The 1.184 kcal/mol and 0.592 kcal/mol thresholds were chosen because they correspond to 2 and 1 kT levels of thermal fluctuation, respectively. The positives in the dataset are thus formed by 677 residues with a |∆∆G_binding_| ≥ 2 kcal/mol; 1,121 residues with |∆∆G_binding_| ≥ 1.184 kcal/mol; and 1,675 with |∆∆G_binding_| ≥ 0.592 kcal/mol. The remaining residues are considered as null spots and form the negatives in the dataset. Table 1 summarizes the number of mutations retained (positives and negatives) in function of the |∆∆G_binding_| threshold value.

**TABLE 1:** Number and percentage of total positive and negative residues retained in function of the |∆∆G_binding_| threshold.

|∆∆G_binding_| threshold (kcal/mol)	Number of positive residues (“significant”)	Percentage of positives (%)	Number of negative residues (“insignificant”)	Percentage of negatives (%)	Total number of residues
2	667	21.9	2,372	78.1	3,039
1.184	1,121	36.9	1,918	63.1	3,039
0.592	1,675	55.1	1,364	44.9	3,039

Chain A of PDB 5DWU was removed from residue 107 onward because there were too many missing residues in the structure, which led to disconnected RINs and errors in the RCA calculation. We also removed the last residue (125) in chain A of 1H9D for the same reason.

### Residue Interaction Network Generation

Residue interaction networks were generated for each PDB file of the SKEMPI 2 dataset (wild-type structures) with in-house software written in C. Contacts (edges) were generated if any interatomic distance between two residues lay between 2.5 Å and 5 Å, ignoring crystallographic water molecules. All the residues of all the chains in a PDB file were considered for contact detection, including both intra-chain and inter-chain contacts; thus, we did not focus on the interface only for RIN generation but considered the overall structure of individual complexes.

A second set of RINs was generated with water molecules, where a contact (edge) was created if the distance from the oxygen atom to an amino acid lay between 2.5 Å and 3.5 Å. All water molecules in a PDB were considered for the RIN generation. In case multiple contacts were detected between the same two residues or between the same residue/water pair, only the shortest distance was used.

### Centralities

We considered 6 different centralities that were run with the same in-house software, which employs the igraph library (Csárdi and Nepusz, 2006):

(1) BCA for betweenness centrality analysis
(2) CCA for closeness centrality analysis
(3) RCA for residue centrality analysis as defined by del Sol et al. (2006)
(4) ECA for eigenvector centrality analysis
(5) DCA for degree centrality analysis
(6) PRA for PageRank analysis as defined by Brin and Page (1998)

For each of these, a Z-score per node (*Z*_*k*_) was calculated like in Brysbaert et al. (2017): $Z_{k}=\frac{C_{k}-\bar{C}}{\sigma }$ , where k is a node, *C* is the centrality, $\bar{C}$ is its average, and *σ* is the corresponding standard deviation; except for RCA for which the Z-score is defined as: $Z_{k}=\frac{\Delta L_{k}-\bar{\Delta L}}{\sigma }$ , where k is a node, $\Delta L_{k}$ is the change of the average shortest path length when node k is removed, $\bar{\Delta L}$ is the corresponding average, and *σ* is the corresponding standard deviation.

We considered the residues with a Z-score ≥ 2 as central. We also optionally considered a weight based on the residue–residue and water–residue distances in order to integrate them into the calculation of centralities. Seven formulas were evaluated, which are as follows:

(1) $\text{W}=\frac{1}{1+{\left(\frac{r-r0}{r1-r0}\right)}^{2}}$ (adapted from Basu and Wallner (2016))
(2) $\text{W}=\frac{1}{r}$ (from RINalyzer (Doncheva et al., 2011) and NAPS (Chakrabarty and Parekh, 2016))
(3) $\text{W}=\frac{1}{1+\left(\frac{r-r0}{r1-r0}\right)}$ (adapted from Basu and Wallner (2016))
(4) $\text{W}=\frac{r+r0-2*r1}{2*\left(r0-r1\right)}$ (linear function from 1(r = *r*_0_) to 0.5 (r = *r*_1_))
(5) $\text{W}=\frac{1}{1+r}$ (adapted from weight formulas 2 and 3)
(6) $\text{W}={\text{r}}_{\text{max}}+1-\text{r}$ (from RINalyzer (Doncheva et al., 2011))
(7) $\text{W}=\frac{r\max^{2}+1-r^{2}}{r\max^{2}+1}$ (adapted from RINalyzer (Doncheva et al., 2011))

where *r* is the residue–residue or residue–water distance, r0 is the minimum detection threshold, r1 is the maximum detection threshold, and r_max_ is the maximum detected distance in the RIN.

To evaluate the capacity of each centrality measure to identify major residues in binding, we computed true positives (TP), true negatives (TN), false positives (FP), and false negatives (FN) as follows:

- TP = card{|∆∆G_binding_| ≥ *threshold* and Z_centrality_ ≥ 2}
- TN = card{|∆∆G_binding_| < *threshold* and Z_centrality_ < 2}
- FP = card{|∆∆G_binding_| < *threshold* and Z_centrality_ ≥ 2}
- FN = card{|∆∆G_binding_| ≥ *threshold* and Z_centrality_ < 2}

where “card” stands for the cardinality or the number of elements in the set.

We further computed the following:

- PPV (positive predictive value, also known as precision) = $\text{PPV}\;\frac{TP}{TP+FP}$

- NPV (negative predictive value) = $\frac{TN}{TN+FN}$

- Sensitivity (also known as recall) = $\frac{TP}{TP+FN}$

- Specificity = $\frac{TN}{TN+FP}$

- Accuracy = $\frac{TP+TN}{TP+TN+FP+FN}$

We also computed the F1 score as follows:

$$F1=2*\frac{precision*recall}{precision+recall}.$$

The “venn” 1.9 R package was used to draw the Venn diagrams. The “ggplot2” 3.3.2 (Wickham, 2016) and “precrec” 0.12.5 (^2^) R packages were used to draw the precision–recall curves.

### Location

For each mutation, we kept the annotation of the residue in the SKEMPI 2 dataset, which follows Levy’s scheme (Levy, 2010): COR for core (buried upon binding and mostly exposed to solvent when unbound), RIM (partly buried residues upon binding), SUP for support (entirely buried when binding and mostly buried when unbound), SUR for surface residues, and INT for interior, with the two last categories representing residues away from the binding site.

### Availability of Data

The files used and generated for this work are available on the git repository: https://gitlab.in2p3.fr/cmsb-public/rin-ppi.

## Results

### Generation of Residue Interaction Networks and Centrality Runs

For each PDB file of the SKEMPI 2 dataset, we generated a residue interaction network. Then for each RIN, we ran the 6 centrality measures: 3 that are based on shortest paths (BCA, CCA, and RCA) and 3 that are based on local interactions of nodes in the network (ECA, DCA, and PRA). A Z-score was computed for each of them, and a residue was considered as central when its Z-score was superior or equal to 2. We then accumulated the residues that were found as central and that showed a change in the |∆∆G_binding_| superior or equal to a given threshold (true positives), as well as other statistics as described in Materials and Methods. We note here that the main statistics of interest are the precision (PPV) and the sensitivity (recall): the precision because it shows how well a centrality measure manages to properly identify a residue of interest and the sensitivity because it shows the proportion of the residues of interest that were identified. A good prediction associates high precision and high sensitivity, meaning the centrality manages to find the majority of the major residues. Table 2 gives these results for each threshold.

**TABLE 2:** Number of identified residues and associated statistical measures for each centrality, applying a different threshold for |∆∆G_binding_|.

|∆∆G_binding_| ≥ 2 kcal/mol						
	BCA	CCA	RCA	ECA	DCA	PRA
TP	194	74	156	156	61	39
TN	2,066	2,274	2,120	2,153	2,311	2,330
FP	296	88	242	209	51	32
FN	483	603	521	521	616	638
PPV	0.3959	0.4568	0.3920	0.4274	0.5446	0.5493
NPV	0.8105	0.7904	0.8027	0.8052	0.7895	0.7850
Sensitivity	0.2866	0.1093	0.2304	0.2304	0.0901	0.0576
Specificity	0.8747	0.9627	0.8975	0.9115	0.9784	0.9865
Accuracy	0.7437	0.7726	0.7489	0.7598	0.7805	0.7795
**|∆∆G_binding_| ≥ 1.184 kcal/mol**						
	BCA	CCA	RCA	ECA	DCA	PRA
TP	291	118	234	229	83	51
TN	1,719	1,874	1,754	1,782	1,889	1,898
FP	199	44	164	136	29	20
FN	830	1,003	887	892	1,038	1,070
PPV	0.5939	0.7284	0.5879	0.6274	0.7411	0.7183
NPV	0.6744	0.6514	0.6641	0.6664	0.6454	0.6395
Sensitivity	0.2596	0.1053	0.2087	0.2043	0.0740	0.0455
Specificity	0.8962	0.9771	0.9145	0.9291	0.9849	0.9896
Accuracy	0.6614	0.6555	0.6542	0.6617	0.6489	0.6413
**|∆∆G_binding_| ≥ 0.592 kcal/mol**						
	BCA	CCA	RCA	ECA	DCA	PRA
TP	366	140	298	287	97	57
TN	1,240	1,342	1,264	1,286	1,349	1,350
FP	124	22	100	78	15	14
FN	1,309	1,535	1,377	1,388	1,578	1,618
PPV	0.7469	0.8642	0.7487	0.7863	0.8661	0.8028
NPV	0.4865	0.4665	0.4786	0.4809	0.4609	0.4549
Sensitivity	0.2185	0.0836	0.1779	0.1713	0.0579	0.0340
Specificity	0.9091	0.9839	0.9267	0.9428	0.9890	0.9897
Accuracy	0.5285	0.4877	0.5140	0.5176	0.4758	0.4630

It shows that the accuracy of each measure increases when the free-energy difference threshold is raised. This can be explained by the fact that the measures generally succeed in identifying the nonessential residues better than the residues of interest, keeping specificity very high compared to sensitivity. The residues of interest, on the other hand, are better identified when the threshold is decreased to the lower value of 0.592 kcal/mol since the PPV increases to more than 74% for all centralities. In essence, it shows that the centralities identify residues that are not necessarily so-called hot spots but still play a nonnegligible role in the interaction. Consequently, the remainder of the article focuses primarily on the results using the lower 0.592 kcal/mol threshold, but all results are available in Supplementary Materials if not present in the main article.

It is noticeable that BCA, ECA, and RCA find many more true positives than CCA, DCA, or PRA. Of the three centralities that are all based on shortest path lengths, CCA gives a lower number of true positives, but its PPV is systematically higher. Of the other centralities, DCA and PRA show a low amount of true positives but their PPV are the highest of them all, culminating at 0.87 for DCA when considering the |∆∆G_binding_| threshold of 0.592 kcal/mol.

The sensitivity of every measure can be considered low. Its best value of 0.29 is found for BCA when considering the highest |∆∆G_binding_| threshold. This means that many of the residues of interest fail to be detected by the centrality measures. However, the relatively high precision for the 0.592 kcal/mol threshold shows that whenever a residue is identified as central, it is often a residue that is relevant to the binding.

**FIGURE 1:**
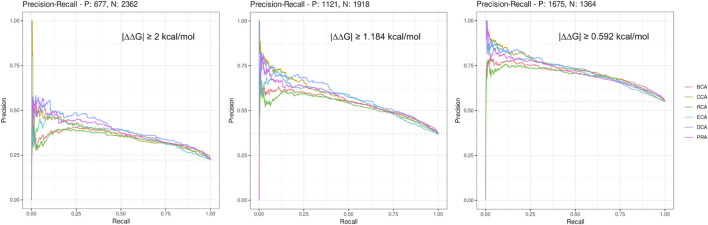
Precision–recall curves drawn for each centrality measure for the 3 thresholds of |∆∆G_binding_|.

Here, we considered a residue as central if its Z-score ≥ 2, a definition commonly used for the identification of central residues. In order to get a broader view, we drew the precision–recall curve of each centrality measure for the 3 thresholds of |∆∆G_binding_| (see Figure 1). The graphs show that at low recall, the precision is very high (≥ 0.75) for the lowest |∆∆G_binding_| and then decreases smoothly, staying always superior to 0.5 even at maximum sensitivity. The shapes of all curves are similar but precision is lowered for higher |∆∆G_binding_| thresholds. The most precise centralities are CCA, DCA, and PRA at low recall but DCA shows better results at slightly higher recall, for every |∆∆G_binding_| threshold. BCA and RCA are less precise, whereas ECA shows results comparable to those of the 3 most precise centralities, for the lowest threshold of |∆∆G_binding_|. In conclusion, increasing the Z-score threshold, which reduces the sensitivity, allows to increase the precision to very high levels, especially when considering the lowest threshold for |∆∆G_binding_|. The commonly used threshold of 2 for Z-scores looks reasonable considering these precision–recall curves.

### Water Molecules Increase Sensitivity

We initiated our investigation into the role of water molecules in residue interaction networks a few years ago (Brysbaert et al., 2018). We then showed that the inclusion of water molecules in RINs increased the number of central residues found in the 2 complexes treated. Here, we address the same question, namely, whether it is preferable to consider water molecules in centrality analysis, but now applied to the same selection of the SKEMPI 2 dataset as before, computing the exact same statistics.

**FIGURE 2:**
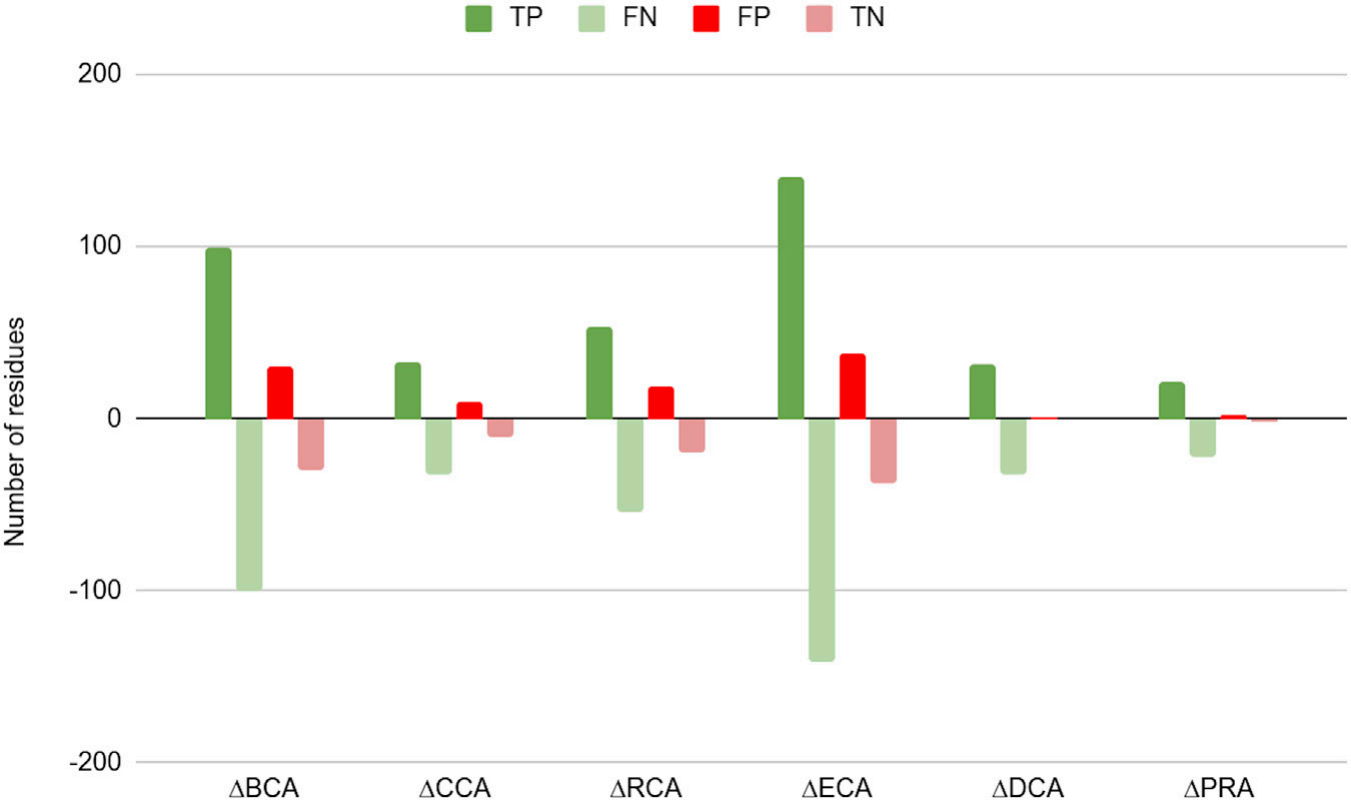
Change in number of true positives (TP), false negatives (FN), false positives (FP), and true negatives (TN) for the 6 centrality measures run on residue interaction networks (RINs) with water versus those without water; a threshold of 0.592 kcal/mol was considered for |∆∆G_binding_|.

Figure 2 shows the difference in number of residues found as TP, TN, FP, and FN between results with water and without water for |∆∆G_binding_| ≥ 0.592 kcal/mol (the results for the 2 other thresholds are available in Supplementary Figure S1 and Supplementary Table S1). For all centrality measures, the number of positives increased, both true as well as false positives. As a consequence, the number of negatives decreased to the same extent. The shift is marginal for CCA, DCA, and PRA, which can be explained by the low number of central residues identified by these measures.

This shift in categories is due to a global rise of Z-scores for protein residues in the presence of water in the network, an effect already shown in Brysbaert et al. (2018). This raises the number of true positives but also that of false positives. This makes the PPV to change slightly upon inclusion of water in the networks, although it is slightly lowered for PRA (for the higher thresholds for |∆∆G_binding_|, see Table 2, and Supplementary Table S1), likely due to the fact that this centrality shows the lowest number of true positives. The sensitivity, however, is increased for all centralities on water-containing networks, gaining up to 11.67% for ECA for the |∆∆G_binding_| ≥ 2 kcal/mol threshold. The specificity is found to decrease slightly with accuracy generally increasing, especially for the lowest threshold for |∆∆G_binding_|.

In conclusion, by including water in the networks, the centralities overall find the same ratio of relevant residues among all detected central residues (precision), but the absolute number of these is higher (sensitivity). It is therefore recommended to include water molecules in RIN generation, confirming our previous study of the colicin E2 DNase–Im2 and barnase/barstar complexes (for any threshold of |∆∆G_binding_|). For the specific case of PRA, which shows fewer results but with higher precision (specifically for the higher |∆∆G_binding_| thresholds), we advise investigating and comparing centralities with and without water, notably because this PageRank shows the lowest sensitivity of all measures.

### Weighting Distances Increases Sensitivity for Eigenvector Centrality Analysis

**FIGURE 3:**
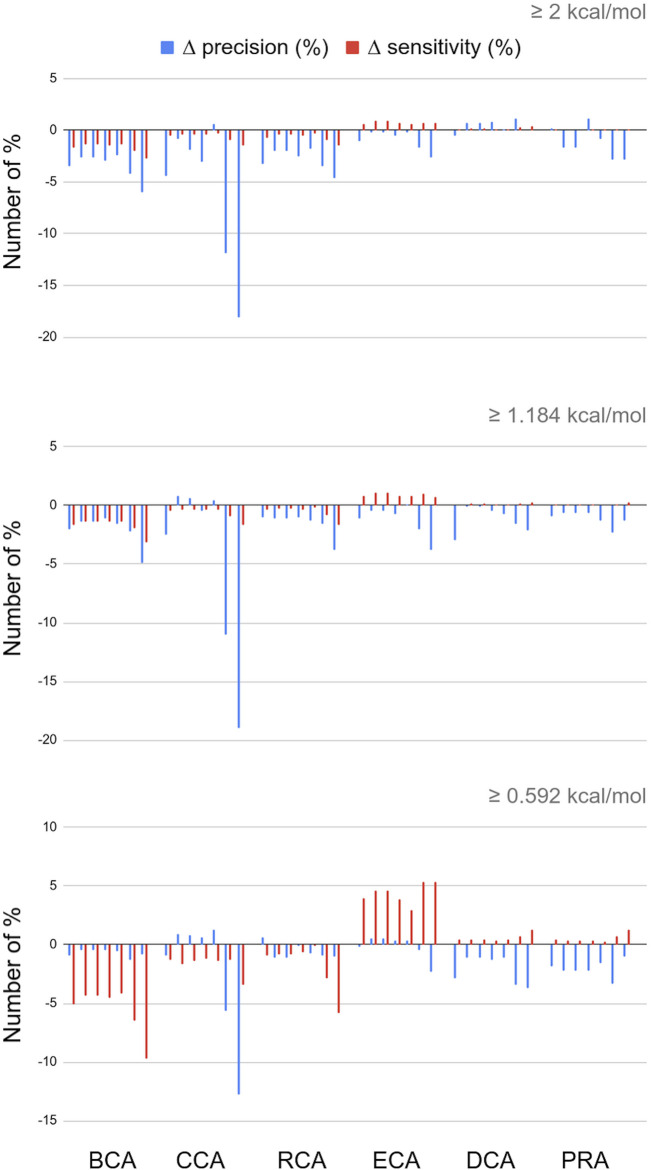
Differences in percent numbers for precision and sensitivity between weighted and unweighted centralities; water molecules are included in the networks; for every centrality calculation, the 7 bars shown correspond to the weight formulas 1 through 7.

The RINs were generated including contacts in a binary mode: it is only when the residue–residue contact distance was between 2.5 Å and 5 Å, that the two corresponding nodes were connected via an edge (similar for water–residue contacts between 2.5 Å and 3.5 Å). These distances could be used to weight the edges in the RINs. Thus, we tested 7 formulas of edge weights and calculated the centralities (see Materials and Methods for formulas). Figure 3 presents the difference (in number of %) of the precision and sensitivity between weighted and unweighted centralities for all 7 weights, for each centrality, and always with water. The complete results for the 7 weights for RINs with and without water and for the three thresholds are available in Supplementary Materials in a single spreadsheet.

For the three global centrality measures BCA, CCA, and RCA, weighting the distances worsens the results, whatever the threshold for |∆∆G_binding_|. Indeed, integrating any weight calculation lowers the number of true positives. Despite a lower number of false positives, precision and sensitivity also decreased, varying with the specific weight formula and threshold for |∆∆G_binding_|.

For the other measures, however, the number of true positives increases, but the number of false positives increases as well. With the occasional exception, the overall precision is unchanged or lowered upon the inclusion of edge weights. In the case of DCA and PRA, the sensitivity values are so low that even small changes in TP, TN, FP, and FN can have a large impact on the statistics. Integrating weights in these centrality calculations is therefore not recommended.

The only constant that we observe is that ECA wins in sensitivity whatever the weight formula and |∆∆G_binding_| threshold, up to 5.91% for weight formula 7 with water. The disadvantage is that this increase in sensitivity is often accompanied by a decrease in precision. It is interesting to note, however, that this decrease in precision is limited when water molecules are included in the network (see Supplementary Materials), and this is applicable to any threshold for |∆∆G_binding_|. The effect is observed for all weight formulas and is optimal for weight formula 5. Weight formulas 6 and 7, which are based on a subtraction of the distance from a reference value, show the highest increase in sensitivity but at a general cost of precision.

In conclusion, using any 1 of the first 5 weight formulas is essentially interesting for ECA in the presence of water because it enables an increase in sensitivity without significant loss of precision. The best compromise in these is weight formula 5, which shows a slightly smaller increase in sensitivity but with little to no loss of precision, regardless of the threshold for |∆∆G_binding_|.

### Combining Centralities Improves Precision or Sensitivity

**FIGURE 4:**
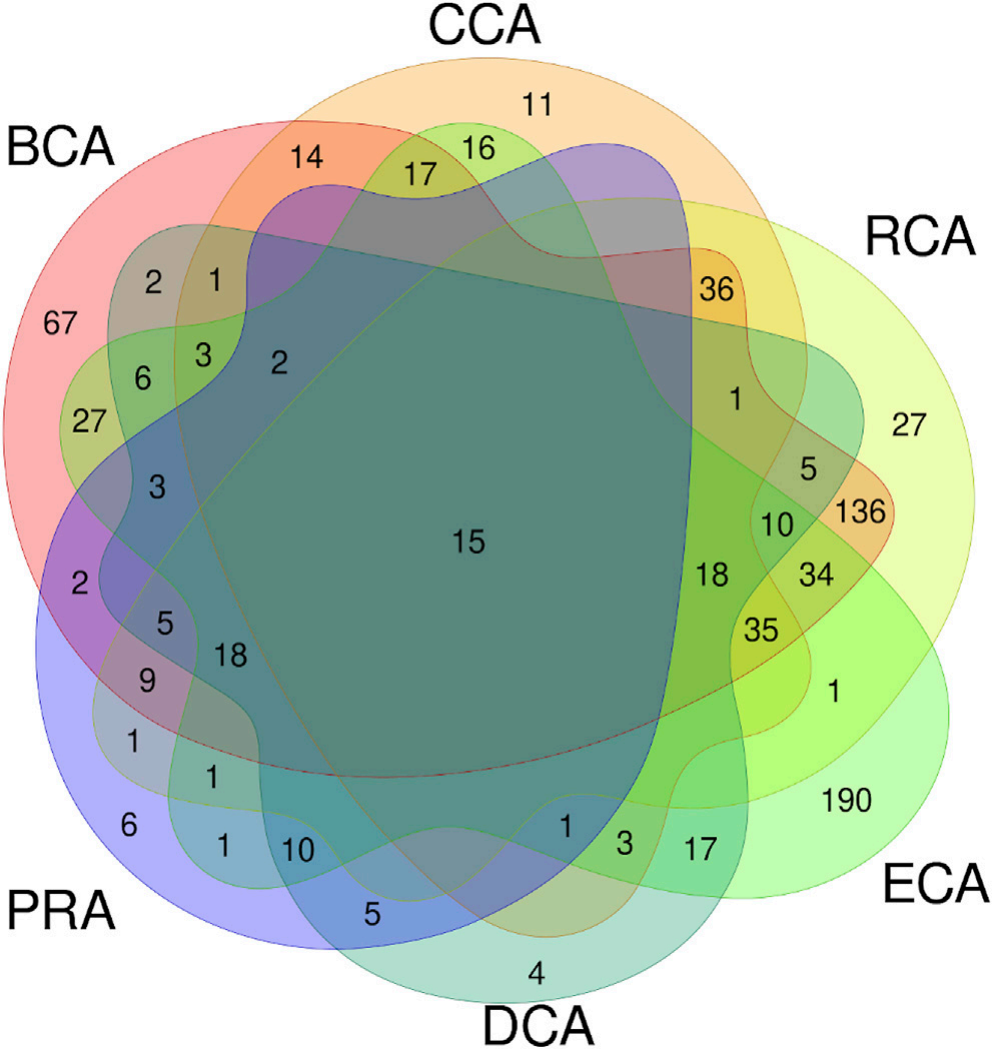
Venn diagram of true positives found for all the 6 centralities performed on residue interaction networks considering water molecules and unweighted edges for a |∆∆G_binding_| ≥ 0.592 kcal/mol threshold.

These 6 centrality measures give heterogeneous amounts of true positives: CCA, DCA, and PRA are the lowest and BCA, RCA, and ECA are the highest numbers, of which BCA is the highest. We asked ourselves if the residues identified were the same among the various measures or if the results were complementary. Figure 4 shows the Venn diagram of the true positives, considering all centralities using unweighted graphs, with water molecules, and for a |∆∆G_binding_| ≥ 0.592 kcal/mol threshold. This diagram clearly shows that a significant amount of true positives are found by several measures but it also shows that some are only found by a single one. In particular, BCA and ECA are able to identify many residues not found by any other measure. RCA shows the largest amount of TP that are also identified by BCA. To evaluate the relevance of combining centralities, we computed all combinations of intersection and all combinations of union of the 6 measures to see if a specific one or a combination of several allowed to raise the precision and/or sensitivity.

**FIGURE 5:**
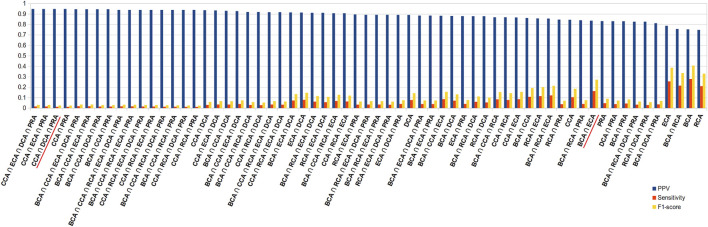
Diagram of precision (blue), sensitivity (red), and F1 score (yellow), ordered in descending order of precision, for all combinations of intersections of the 6 centrality measures performed on residue interaction networks considering water molecules and unweighted edges for a |∆∆G_binding_| ≥ 0.592 kcal/mol threshold; combinations cited in the text are underlined in red.

Because of the added value of including water molecules, we further investigated these, but continued with unweighted centrality calculations. Intersection between many measures should increase the precision with a decrease in sensitivity. Figure 5 shows the precision, sensitivity, and F1 score in function of all combinations of intersections, arranged in descending order of precision. This diagram indeed shows that intersecting central residue sets found by multiple measures and even going up to all 6 increases the precision to maximum or close to maximum. The highest values are obtained for the intersections that involve CCA, DCA, and PRA but at the cost of poor sensitivity. A large number of combinations of intersections show very close precisions; thus, in order to limit the loss of sensitivity but keep precision superior to 80%, the best choice is certainly BCA ∩ ECA, which has the highest F1 score compared to all other combinations for such a level of precision. Results for the 2 other thresholds are shown in Supplementary Figure S2; they show the same tendencies, including the fact that BCA ∩ ECA shows the best compromise.

**FIGURE 6:**
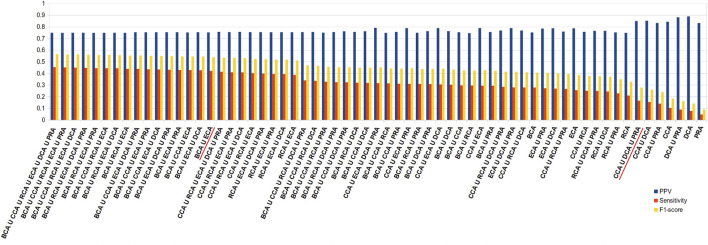
Diagram of precision (blue), sensitivity (red), and F1 score (yellow), ordered in descending order of sensitivity, for all combinations of unions of the 6 centrality measures performed on residue interaction networks considering water molecules and unweighted edges for a |∆∆G_binding_| ≥ 0.592 kcal/mol threshold; combinations cited in the text are underlined in red.

In order to improve the sensitivity, the union of centralities should be considered. We ran the same calculations, this time trying all the combinations of unions of centralities. Figure 6 shows the results like Figure 5 but for unions instead of intersections. Here, the results are arranged in descending order of sensitivity. As observed in the intersections, the higher the sensitivity, the lower the precision, but here the loss in precision is less drastic upon an increase in sensitivity. Therefore, while intersecting the sets of central residues from the 6 centralities gives among the highest rates of precisions, the union of all gives the highest sensitivity (0.454) for precision that stays relatively high (0.749). Nevertheless, the results are quite stable for many combinations of unions and in the neighborhood of the union of all 6 measures. Therefore, considering only BCA ∪ ECA provides the close to best results, even if precision is unchanged with respect to both measures individually (equal for BCA and −0.035 for ECA), sensitivity increases significantly ( + 0.143 for BCA and + 0.166 for ECA). It should also be noted that because CCA, DCA, and PRA all individually show precision superior to 0.8, considering the union of these 3 keeps precision at that level (0.850) while doubling the sensitivity (0.166) with the highest F1 score for such a level of precision. Results are similar for the other thresholds of |∆∆G_binding_| (see Supplementary Figure S3).

Finally, we considered all the same parameters for RIN generation and centrality analysis except for ECA, which we weighted using weight formula 5. Supplementary Table S2 shows the difference in precision and sensitivity when using weight formula 5 for ECA or not. When considering unions with weighted ECA (Supplementary Table S2A), the precision does not significantly change, while sensitivity increases by up to 0.032 (+0.023 in the case of BCA ∪ ECA). For intersections (Supplementary Table S2B), the results are relatively unchanged, sometimes a bit lower, but BCA ∩ ECA allows an increase of 0.016 in precision and 0.014 in sensitivity. The intersection of all the measures shows a very low decrease in precision and no change in sensitivity (−0.004 and 0, respectively). These results recommend the use of edge weights for ECA, particularly in the case of the union with other centrality measures.

Altogether, these results show that if a high precision is required, taking the intersection of the 6 centralities is the best solution, while if the highest number of true positives needs to be found, that is, a high sensitivity, the union of these centralities is the best. Nevertheless, subsets of centralities can lead to good results, such as CCA ∩ DCA ∩ PRA for high precision or CCA ∪ DCA ∪ PRA for slightly lower precision, but with higher sensitivity. The results for BCA ∩ ECA are close to the latter and could be run in parallel since they do not necessarily highlight the same residues. For a high sensitivity at reduced effort, the results for BCA ∪ ECA are comparable to the union of the 6 centralities. Furthermore, weighted edges in ECA allow for some increase in sensitivity.

### Centrality Measures Locate Relevant Residues in Different Structural Regions

We further investigated the capacity of each measure to find crucial residues in 5 structural regions as defined by Levy (2010) and provided in the SKEMPI 2 dataset. Table 3 shows the location of the TP found for each measure with or without water and also with weight formula 5 for ECA, for a threshold of |∆∆G_binding_| ≥ 0.592 kcal/mol. The tendencies are the same for the 2 other thresholds (data not shown).

**TABLE 3:** Number of true positives found per centrality per region; the total number of residues of interest per region (with |∆∆G_binding_| ≥ 0.592 kcal/mol upon mutation) is written behind the slash and the proportion is calculated for each; (W) means that water is included and the associated columns have a blue background; (W5) means that water is included and weight formula 5 is used and its column has a green background.

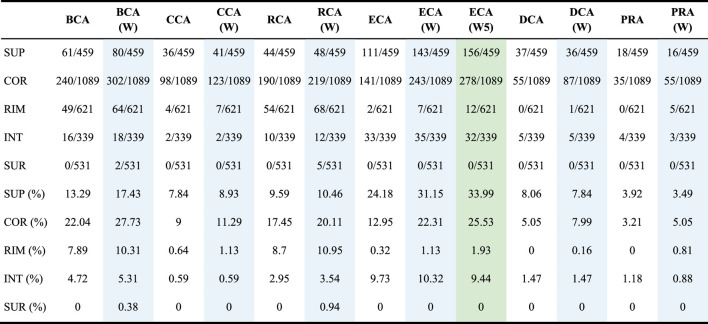

The table shows that all the measures preferentially find central residues at the interface: in the SUP (support) and COR (core) regions, followed by RIM, and then in the INT (interior) region which is far away from the interface. The SUR (surface) region is not favored, but the inclusion of water has an effect of finding a few of them with BCA and RCA. Of all centrality measures, ECA is the one that finds the highest number of SUP residues, while BCA finds the highest number of COR residues. For RIM, BCA and RCA are the best, while ECA is the best for INT. Adding water has the effect of increasing the number of residues in each category. The use of a weight for ECA has the same effect. The 3 measures that show a low sensitivity but a high precision, which are CCA, DCA, and PRA, essentially focus on SUP and COR residues.

Nonetheless, even if a measure performs better in identifying major residues in a specific location, other centralities may still find some that were not identified by the preferred measure. Consequently, the choice of the best centrality measure to identify central residues depends on the structural area of interest but in any case, considering intersections or unions is advised in order to improve the precision through the former or the sensitivity through the latter.

## Discussion

**FIGURE 7:**
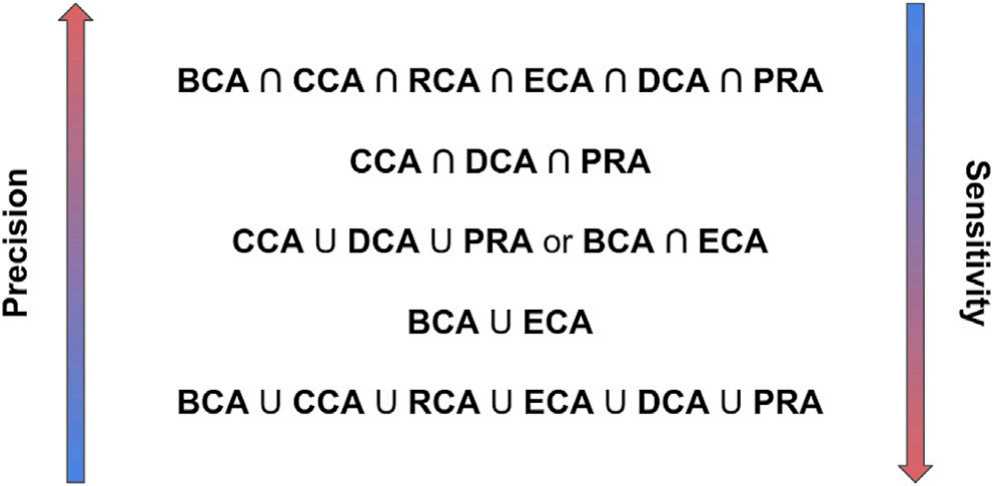
Recommended combinations of centrality analyses to identify major binding residues from highest precision to highest sensitivity.

Identifying the major residues in the binding of 2 or more proteins is a crucial task to understand their function, plan mutagenesis experiments, or target drug design. Major residues are understood here as residues which enhance or weaken an interaction upon mutation. We evaluated the capability of 6 centrality measures to identify these major residues in residue interaction networks generated from the three-dimensional structures of complexes. Our results demonstrate that the CCA, PRA, and DCA show a high precision, that is, have the highest probability that these residues are in fact major binding residues, for any threshold of |∆∆G_binding_| considered, but a low sensitivity. In contrast, BCA, RCA, and ECA show a higher sensitivity, that is, they find the maximum number of major binding residues, with lower precision. Including water increases the sensitivity without losing precision, while integrating a weight in centrality computation increases sensitivity for ECA. Taking the intersection of several centralities improves the precision, the highest precision being obtained for the intersection of the results of the 6 centralities but with lowered sensitivity. Unions increase sensitivity at the cost of precision, with the highest sensitivity found for the union of the 6 centralities. However, when searching for relevant binding residues, we suggest proceeding step-by-step using a combination of measures. The intersection of selected centralities favors identification with highest precision, while the union favors sensitivity. Figure 7 presents this step-by-step approach that we propose from our results.

While RCA has shown its merit (Hu et al., 2014; de Ruyck et al., 2018; Laulumaa et al., 2018), it is not the most important one in a combined approach for the identification of relevant binding residues. This is mainly due to the fact that its ensemble of identified central residues is in a large part included in the ensemble of central residues found by BCA. Nevertheless, depending on the network, RCA can be preferred, for example, for the unfavorable case of disconnected networks to which BCA is more sensitive (Brysbaert et al., 2019). Related to this, the inclusion of water molecules also has the effect of limiting disconnections in an RIN.

Our work shows that a combination of centrality measures, or even individual ones, can lead to good precision. However, even in the best cases, with the union of all centralities, the sensitivity hardly exceeds 45%. This means that these measures will never find all the residues of interest, except lowering the Z-score threshold which would lead to a loss in precision. Nonetheless, one should realize that in reality, one rarely wants to find them all. What is really needed is what the precision reveals, namely, that whenever a residue is identified as central, this residue is indeed relevant to the binding. For instance, for mutagenesis experiments or drug design, the identification of anywhere between 1 to 5 major binding residues is already of great value, and that is something that the centrality measures evaluated in this article can perform, thereby proving they are an efficient tool to identify major binding residues.

The SKEMPI 2 list is obviously not exhaustive and the structures therein most probably contain other major binding residues. It is thus possible that some residues identified as central by 1 or several of the 6 measures are major binding residues that are not listed in the SKEMPI 2 dataset. An exhaustive knowledge of all these disrupting residues would make the evaluation more accurate. Nonetheless, the set of 3,039 residues is of sufficient size to provide correct statistics.

The precision for the |∆∆G_binding_| ≥ 2 kcal/mol threshold is not very good, lying between 38 and 55% depending on the centrality measure and inclusion of water. Nevertheless, the number of mutations leading to such a disruption in binding is roughly one-fifth of the 3,039 residues (21.9%). When the proportions between positives and negatives are almost equal, which is the case with the lowest threshold of |∆∆G_binding_| ≥ 0.592 kcal/mol (55% positives), all measures achieve good precision (between 74 and 89%). This means that a majority of the residues that are found as false positives when considering the highest threshold for |∆∆G_binding_| are residues that still show a |∆∆G_binding_| ≥ 0.592 kcal/mol upon mutation and as such, are residues that do disrupt the binding when mutated.

Our construction strategy of the RINs was based on distances between all heavy atoms in the structure, setting an edge between 2 residues if an interatomic distance was between 2.5 Å and 5 Å. We purposely kept this definition simple to put the emphasis on the evaluation of centrality measures. It is also the definition used in CASP and CAPRI to calculate inter-chain residue–residue contacts (Lensink et al., 2019; Lensink et al., 2020). We are aware that the manner to generate the RINs may have an effect on the centrality results although Faisal et al. (2017) showed, for instance, that choosing a threshold of 4 Å, 5 Å, or 6 Å between any heavy atom or 7.5 Å between ∝-carbon has a minimal effect on protein structural comparison accuracy. Generating various types of residue interaction networks by considering different definitions for creating a contact between two residues, for example, as done by the RING program (Piovesan et al., 2016) in function of the type of interaction or through more sophisticated methods such as Voronoi tessellation (Cazals et al., 2006; Olechnovič and Venclovas, 2019), might be an interesting future avenue of investigation.

We finally checked the location of the major binding residues identified by the 6 measures. We showed that the measures do not systematically identify residues in the same region of the structure. This correlates well with the fact that the sets of central residues do not fully cover each other. Therefore, the choice of a certain centrality measure to identify major binding residues may depend on the area of research. In any case, combining different measures is still a preferred approach. It is interesting to note that the centralities find up to 10% of the INT residues (for ECA), which are interior residues and thus not easy to identify as they are not directly connected to the interface. The advantage of RINs here is that we work with a network that represents the whole complex and therefore may identify residues that are indirectly involved in the binding. However, the surface residues, which are also away from the binding site, are most often not identified even if a few are anecdotally found by RCA and BCA, both in the presence of water. This effect may be due to the fact that water adds edges to the network at the surface, essentially moving surface residues inwards. Indeed, SUR residues correspond to nodes at the outer sides of the network, away from the binding site; they possess fewer connections than internal nodes in the network and fewer shortest paths across these nodes. The main locations found for residues of interest are SUP, COR, and RIM, which is not surprising because these are directly connected to the binding region.

To conclude, the 6 measures of centrality we evaluated show good complementary performances to identify residues whose mutation disrupts protein–protein binding. As their purpose is not to identify *all* binding residues, they show excellent precision, but do so with limited sensitivity. The sensitivity can be improved slightly by including water molecules in the networks and using weighted edges for ECA. Combining several centrality measures through intersection or union generally leads to improved precision or improved sensitivity, respectively. We believe that these network-based measures could be further combined in a more advanced way than the simple unions or intersections in a machine learning approach for an improved prediction of major binding residues. For an even better prediction, they could also be integrated as features next to structure-based and sequence-based ones like the PREVAIL tool does for the inference of catalytic residues (Song et al., 2018). In any case, centrality analyses are complementary to a visual inspection of the structure and to other methods to find major binding residues like alanine scanning (Kortemme et al., 2004), hot spot predictions (Moreira et al., 2017), or prediction of mutation effects in 3D structures (Ittisoponpisan et al., 2019).

## Data Availability

Publicly available datasets were analyzed in this study. These data can be found here: https://life.bsc.es/pid/skempi2/

## Footnotes

1 Protein Data Bank: the single global archive for 3D macromolecular structure data | Nucleic Acids Research | Oxford Academic [Internet] [cited 2021 Feb 26]. Available from:https://academic.oup.com/nar/article/47/D1/D520/5144142.

2 Precrec: fast and accurate precision–recall and ROC curve calculations in R | Bioinformatics | Oxford Academic [Internet] [cited 2021 Apr 30]. Available from: https://academic.oup.com/bioinformatics/article/33/1/145/2525681.
