# Supplementary material for: Centrality Measures in Residue Interaction Networks to Highlight Amino Acids in Protein–Protein Binding
Source: Front Bioinform. 2021 Jun 18;1:684970. doi: 10.3389/fbinf.2021.684970 (PMC9581030; doi:10.3389/fbinf.2021.684970)
Supplement: Supplementary file 1 [file DataSheet1.PDF]

Supplementary Material

Figure S1. Change in number of True Positives (TP), False Negatives (FN), False Positives (FP) and True Negatives (TN) for the six centrality measures run on Residue Interaction Networks (RINs) with water *vs* those without water; **(A)** a threshold of 2 kcal/mol was considered for $|\Delta\Delta G_{binding}|$; **(B)** a threshold of 1.184 kcal/mol was considered for $|\Delta\Delta G_{binding}|$.

A

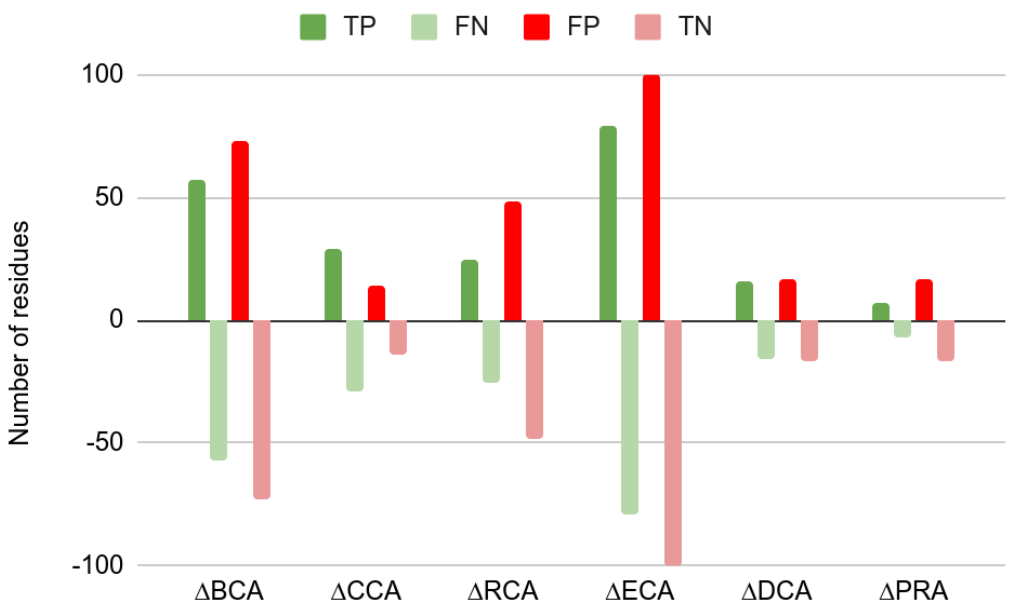

B

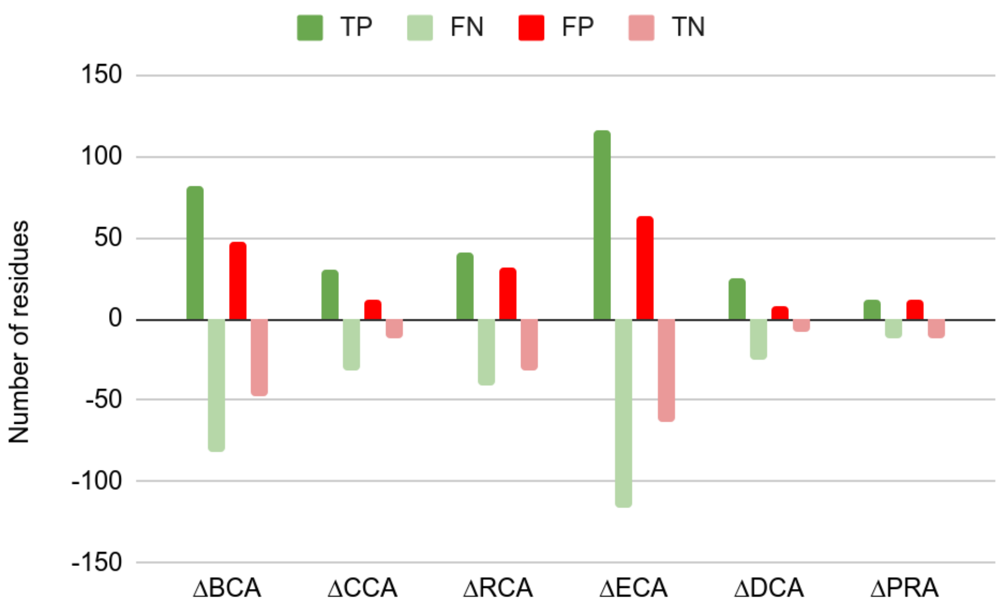

Supplementary Material

Figure S2. Diagram of precision (blue), sensitivity (red) and F1-score (yellow), ordered in descending order of **precision**, for all combinations of **intersections** of the six centrality measures performed on residue interaction networks considering water molecules and unweighted edges, for **(A)** $|\Delta\Delta G_{binding}| \geq 2$ kcal/mol; **(B)** $|\Delta\Delta G_{binding}| \geq 1.184$ kcal/mol

A

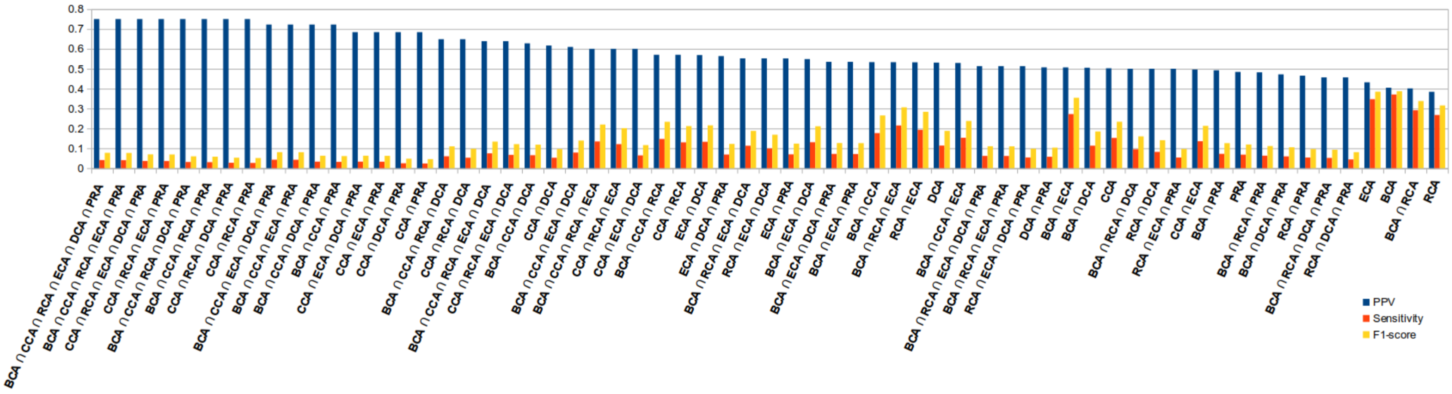

B

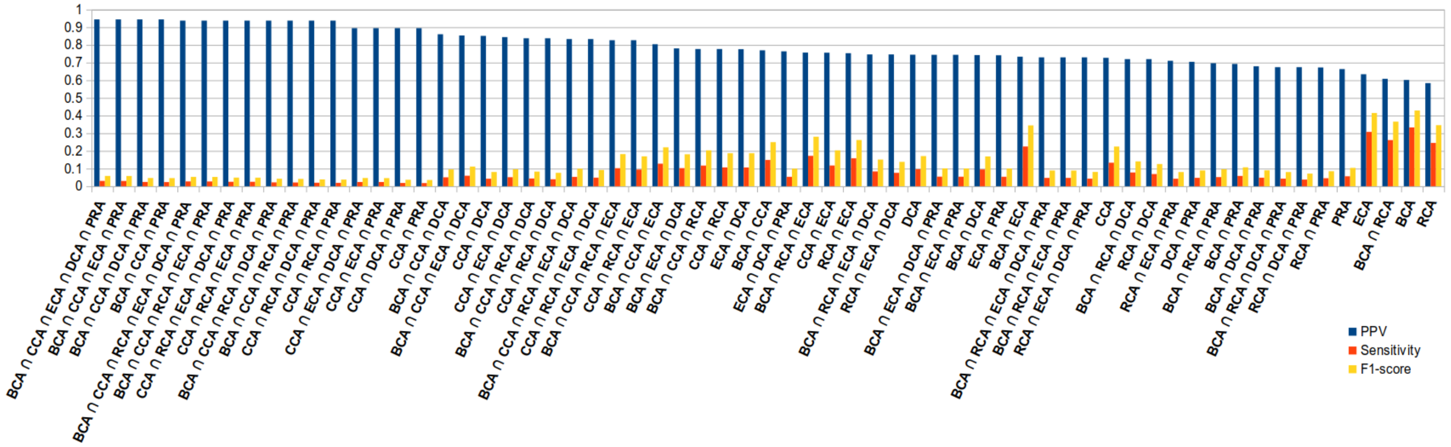

Figure S3. Diagram of precision (blue), sensitivity (red) and F1-score (yellow), ordered in descending order of **sensitivity**, for all combinations of **unions** of the six centrality measures performed on residue interaction networks considering water molecules and unweighted edges, for **(A)** $|\Delta\Delta G_{binding}| \geq 2$ kcal/mol; **(B)** $|\Delta\Delta G_{binding}| \geq 1.184$ kcal/mol

A

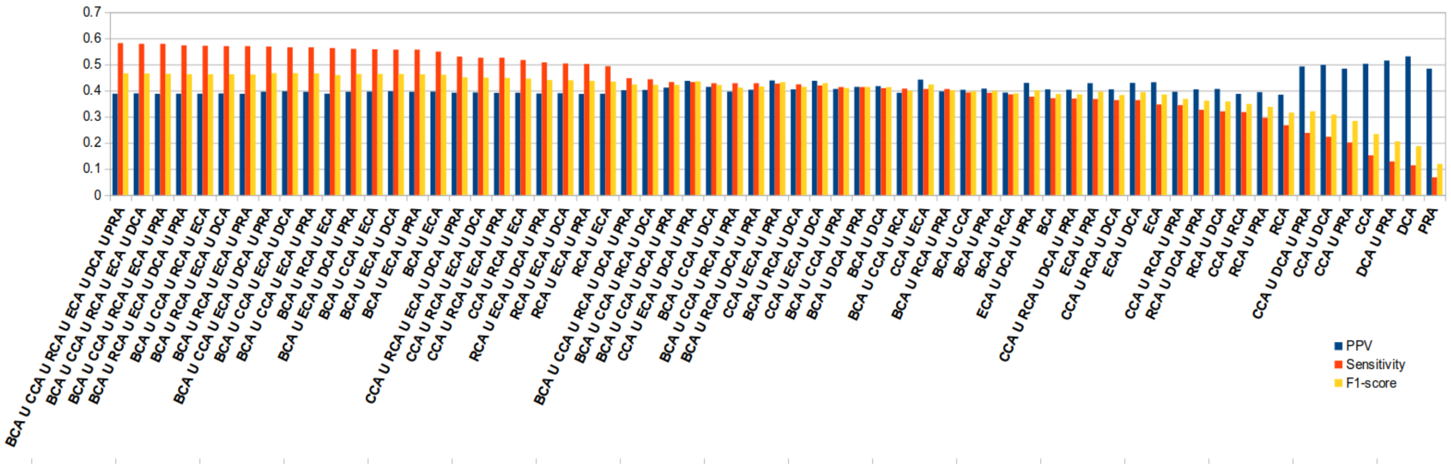

B

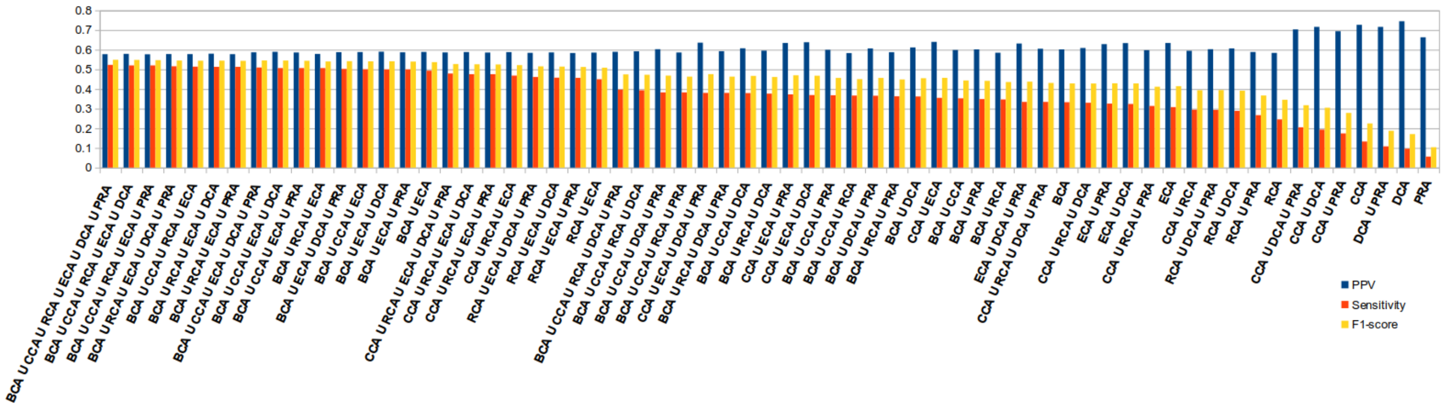

Supplementary Material

Table S1. Results for all centralities, including water molecules in the RINs.

$|\Delta\Delta G_{binding}| \geq 2$ kcal/mol

	BCA	CCA	RCA	ECA	DCA	PRA
TP	251	103	181	235	77	46
TN	1993	2260	2072	2053	2294	2313
FP	369	102	290	309	68	49
FN	426	574	496	442	600	631
PPV	0.4048	0.5024	0.3843	0.4320	0.5310	0.4842
NPV	0.8239	0.7975	0.8069	0.8228	0.7927	0.7857
Sensitivity	0.3708	0.1521	0.2674	0.3471	0.1137	0.0679
Specificity	0.8438	0.9568	0.8772	0.8692	0.9712	0.9793
Accuracy	0.7384	0.7776	0.7414	0.7529	0.7802	0.7762

$|\Delta\Delta G_{binding}| \geq 1.184$ kcal/mol

	BCA	CCA	RCA	ECA	DCA	PRA
TP	373	149	275	345	108	63
TN	1671	1862	1722	1719	1881	1886
FP	247	56	196	199	37	32
FN	748	972	846	776	1013	1058
PPV	0.6016	0.7268	0.5839	0.6342	0.7448	0.6632
NPV	0.6908	0.6570	0.6706	0.6890	0.6500	0.6406
Sensitivity	0.3327	0.1329	0.2453	0.3078	0.0963	0.0562
Specificity	0.8712	0.9708	0.8978	0.8962	0.9807	0.9833
Accuracy	0.6726	0.6617	0.6571	0.6792	0.6545	0.6413

$|\Delta\Delta G_{binding}| \geq 0.592$ kcal/mol

	BCA	CCA	RCA	ECA	DCA	PRA
TP	466	173	352	428	129	79
TN	1210	1332	1245	1248	1348	1348
FP	154	32	119	116	16	16
FN	1209	1502	1323	1247	1546	1596
PPV	0.7516	0.8439	0.7473	0.7868	0.8897	0.8316
NPV	0.5002	0.4700	0.4848	0.5002	0.4658	0.4579
Sensitivity	0.2782	0.1033	0.2101	0.2555	0.0770	0.0472
Specificity	0.8871	0.9765	0.9128	0.9150	0.9883	0.9883
Accuracy	0.5515	0.4952	0.5255	0.5515	0.4860	0.4696

Table S2. Difference in precision (PPV) and sensitivity (recall) considering weight 5 for ECA or not **(A)** for **union** of centralities, ordered by descending values of **Δrecall**; **(B)** for **intersection** of centralities, ordered by descending values of **ΔPPV**; only combinations with ECA are conserved.

A

Union of centralities	ΔPPV	Δrecall
ECA U PRA	0.003	0.032
ECA U DCA	0.003	0.032
ECA U DCA U PRA	0.002	0.031
ECA	0.003	0.030
CCA U ECA U DCA	0.002	0.026
CCA U ECA U PRA	0.002	0.026
CCA U ECA U DCA U PRA	0.001	0.026
CCA U ECA	0.003	0.025
BCA U ECA U PRA	0.000	0.023
BCA U ECA	0.000	0.023
BCA U ECA U DCA U PRA	-0.001	0.022
BCA U ECA U DCA	-0.001	0.022
RCA U ECA U PRA	-0.001	0.022
RCA U ECA U DCA	-0.001	0.022
BCA U RCA U ECA U PRA	0.000	0.022
BCA U RCA U ECA	0.000	0.022
BCA U CCA U ECA U PRA	0.000	0.022
BCA U CCA U ECA	0.000	0.022
CCA U RCA U ECA U PRA	0.001	0.022
RCA U ECA U DCA U PRA	-0.001	0.022
RCA U ECA	-0.001	0.022
CCA U RCA U ECA	0.001	0.022
CCA U RCA U ECA U DCA U PRA	0.000	0.021
BCA U CCA U RCA U ECA	0.000	0.021
BCA U RCA U ECA U DCA	-0.001	0.021
BCA U CCA U ECA U DCA U PRA	0.000	0.021
CCA U RCA U ECA U DCA	0.000	0.021
BCA U CCA U RCA U ECA U PRA	0.000	0.021
BCA U RCA U ECA U DCA U PRA	-0.001	0.021
BCA U CCA U ECA U DCA	0.000	0.021
BCA U CCA U RCA U ECA U DCA	0.000	0.021
BCA U CCA U RCA U ECA U DCA U PRA	0.000	0.021

B

Intersection of centralities	ΔPPV	Δrecall
RCA ∩ ECA	0.022	0.014
BCA ∩ RCA ∩ ECA	0.021	0.014
BCA ∩ RCA ∩ ECA ∩ PRA	0.017	-0.002
RCA ∩ ECA ∩ PRA	0.017	-0.002
BCA ∩ ECA	0.016	0.014
RCA ∩ ECA ∩ DCA ∩ PRA	0.014	-0.003
BCA ∩ RCA ∩ ECA ∩ DCA ∩ PRA	0.014	-0.003
BCA ∩ ECA ∩ PRA	0.014	-0.002
BCA ∩ ECA ∩ DCA ∩ PRA	0.011	-0.003
BCA ∩ RCA ∩ ECA ∩ DCA	0.010	-0.001
RCA ∩ ECA ∩ DCA	0.010	-0.001
ECA ∩ PRA	0.010	-0.001
ECA ∩ DCA ∩ PRA	0.009	-0.002
BCA ∩ CCA ∩ RCA ∩ ECA	0.009	0.010
CCA ∩ RCA ∩ ECA	0.009	0.009
BCA ∩ ECA ∩ DCA	0.007	-0.001
ECA ∩ DCA	0.006	0.001
ECA	0.003	0.030
BCA ∩ CCA ∩ RCA ∩ ECA ∩ DCA	0.000	0.001
CCA ∩ RCA ∩ ECA ∩ DCA	0.000	0.001
BCA ∩ CCA ∩ ECA	-0.001	0.009
CCA ∩ ECA ∩ DCA	-0.002	0.000
BCA ∩ CCA ∩ ECA ∩ DCA	-0.002	0.000
CCA ∩ ECA ∩ PRA	-0.003	0.000
CCA ∩ ECA ∩ DCA ∩ PRA	-0.003	0.000
BCA ∩ CCA ∩ ECA ∩ PRA	-0.003	0.000
BCA ∩ CCA ∩ ECA ∩ DCA ∩ PRA	-0.003	0.000
CCA ∩ RCA ∩ ECA ∩ PRA	-0.004	0.000
BCA ∩ CCA ∩ RCA ∩ ECA ∩ PRA	-0.004	0.000
CCA ∩ RCA ∩ ECA ∩ DCA ∩ PRA	-0.004	0.000
BCA ∩ CCA ∩ RCA ∩ ECA ∩ DCA ∩ PRA	-0.004	0.000
CCA ∩ ECA	-0.005	0.009
